# Artificial Neural Network-Based System for PET Volume Segmentation

**DOI:** 10.1155/2010/105610

**Published:** 2010-09-26

**Authors:** Mhd Saeed Sharif, Maysam Abbod, Abbes Amira, Habib Zaidi

**Affiliations:** ^1^Department of Electronic and Computer Engineering, School of Engineering and Design, Brunel University, West London, Uxbridge UB8 3PH, UK; ^2^Nanotechnology and Integrated Bioengineering Centre, University of Ulster, County Antrim BT37 0QB, UK; ^3^Division of Nuclear Medicine, Geneva University Hospital, 1211 Geneva, Switzerland; ^4^Geneva Neuroscience Center, Geneva University, 1211 Geneva, Switzerland

## Abstract

Tumour detection, classification, and quantification in positron emission tomography (PET) imaging at early stage of disease are important issues for clinical diagnosis, assessment of response to treatment, and radiotherapy planning. Many techniques have been proposed for segmenting medical imaging data; however, some of the approaches have poor performance, large inaccuracy, and require substantial computation time for analysing large medical volumes. Artificial intelligence (AI) approaches can provide improved accuracy and save decent amount of time. Artificial neural networks (ANNs), as one of the best AI techniques, have the capability to classify and quantify precisely lesions and model the clinical evaluation for a specific problem. This paper presents a novel application of ANNs in the wavelet domain for PET volume segmentation. ANN performance evaluation using different training algorithms in both spatial and wavelet domains with a different number of neurons in the hidden layer is also presented. The best number of neurons in the hidden layer is determined according to the experimental results, which is also stated Levenberg-Marquardt backpropagation training algorithm as the best training approach for the proposed application. The proposed intelligent system results are compared with those obtained using conventional techniques including thresholding and clustering based approaches. Experimental and Monte Carlo simulated PET phantom data sets and clinical PET volumes of nonsmall cell lung cancer patients were utilised to validate the proposed algorithm which has demonstrated promising results.

## 1. Introduction

Medical images can be obtained using various modalities such as positron emission tomography (PET), single-photon emission computed tomography (SPECT), computed tomography (CT), magnetic resonance imaging (MRI), and ultrasound (US). PET is a molecular imaging technique used to probe physiological functions at the molecular level rather than to look at anatomy through the use of trace elements such as carbon, oxygen, and nitrogen which have a high abundance within the human body. PET plays a central role in the management of oncological patients beside the other main components such as diagnosis, staging, treatment, prognosis, and followup. Owing to its high sensitivity and specificity, PET is effective in targeting specific functional or metabolic signatures that may be associated with various diseases. Among all diagnostic and therapeutic procedures, PET is unique in the sense that it is based on molecular and pathophysiological mechanisms and employs radioactively labeled biological molecules as tracers to study the pathophysiology of the tumour in vivo to direct treatment and assess response to therapy. The leading current area of clinical use of PET is in oncology, where ^18^F-fluorodeoxyglucose (FDG) remains the most widely used tracer. FDG-PET has already had a large valuable effect on cancer staging and treatment, and its use in clinical oncology practice continues to evolve [1–5].

The main challenge of PET is its low spatial resolution which results in the so-called partial volume effect. This effect should be reduced to the minimum level, so that the required information can be accurately quantified and extracted from the analysed volume. On the other hand, the increasing number of patient scans beside the widespread application of PET have raised the urgent need for effective volume analysis techniques to aid clinicians in clinical diagnosis and set the proper plan for treatment. Analysing and extracting the proper information from PET volumes can be performed by deploying image segmentation and classification approaches which provide richer information than that obtained directly from qualitative assessment alone performed on the original PET volumes [6]. The need for accurate and fast analysis for medical volume segmentation leads to exploit artificial intelligence (AI) techniques. These include artificial neural networks (ANN), expert systems, robotics, genetic algorithms, intelligent agents, logic programming, fuzzy logic, neurofuzzy, natural language processing, and automatic speech recognition [7, 8].

ANN is one of the powerful AI techniques that has the capability to learn from a set of data and construct weight matrices to represent the learning patterns. ANN has great success in many applications including pattern classification, decision making, forecasting, and adaptive control. Many research studies have been carried out in the medical field utilising ANN for medical image segmentation and classification with different medical imaging modalities. Multilayer perceptron (MLP) neural network (NN) have been used by [9] to identify breast nodule malignancy using sonographic images. A multiple classifier system using five NNs and five sets of texture features extraction for the characterization of hepatic tissue from CT images is presented in [10]. Kohonen self-organizing NN for segmentation and a multilayer backpropagation NN for classification for multispectral MRI images have been used in [11]. Kohonen NN was also used for image segmentation in [12]. Computer-aided diagnostic (CAD) scheme to detect lung nodules using a multiresolution massive training artificial neural network (MTANN) is presented in [13].

The aim of this paper is to develop a robust, efficient PET volume segmentation system using ANN. The proposed system is evaluated using different training algorithms and its performance assessed using different metrics. ANN outputs are also compared with the outputs of conventional approaches including thresholding and clustering using experimental PET phantom studies and clinical volumes of nonsmall cell lung cancer patients.

This paper is organised as follows. Section 2 presents mathematical background for the selected approaches. The materials and methods used are described in Section 3. Experimental results and their discussion are given in section 4, and finally some conclusions are presented in Section 5.

## 2. Mathematical Background

### 2.1. Mathematical Model of a Neuron

ANN is a mathematical model which emulates the activity of biological neural networks in the human brain. It consists of two or several layers each one has many interconnected group of neurons. Each neuron in the ANN has a number of inputs (the input vector *P*) and one output (*Y*). The input vector elements are multiplied by weights *w*_1,1_, *w*_1,2_,…, *w*_1,*R*_, and the weighted values are fed to the summing junction. Their sum is simply the dot product (*W*.*P*) of the single-row matrix *W* and the vector *P*. The neuron has a bias *b*, which is summed with the weighted inputs to form the net input *n*. This sum, *n*, is the argument of the transfer function *f* [14]

(1) $$\begin{array}{c} n=\overset{R}{\underset{i=1}{\sum }}w_{1,i}\cdot p_{i}+b, \end{array}$$

(2) $$\begin{array}{c} Y=f\left(W\cdot P+b\right), \end{array}$$

(3) $$\begin{array}{c} Y\left(j\right)=f\left[\overset{R}{\underset{i=1}{\sum }}w_{1,i}\left(j\right).p_{i}\left(j\right)+b\right]. \end{array}$$

The learning process can be summarized in the following steps: (1) the initial weights are randomly assigned, (2) the neuron is activated by applying inputs vector and desired output (*Y*_*d*_), and (3) calculation of the actual output (*Y*) at iteration *j*=1 as illustrated in (3), where iteration *j* refers to the *j*th training example presented to the neuron. The following step is to update the weights to obtain the output consistent with the training examples, as illustrated in

(4) $$\begin{array}{c} w_{1,i}\left(j+1\right)=w_{1,i}\left(j\right)+\Delta w_{1,i}\left(j\right), \end{array}$$

where Δ*w*_1,*i*_(*j*) is the weight correction at iteration *j*. The weight correction is computed by using the delta rule in

(5) $$\begin{array}{c} \Delta w_{1,i}\left(j\right)=\alpha *p_{i}\left(j\right)*e\left(j\right), \end{array}$$

where *α* is the learning rate and *e*(*j*) is the error which can be given by

(6) $$\begin{array}{c} e\left(j\right)=Y_{d}\left(j\right)-Y\left(j\right). \end{array}$$

Finally, the iteration *j* is increased by one, and the previous two steps are repeated until the convergence is reached.

### 2.2. Thresholding

#### 2.2.1. Hard Thresholding

Thresholding is the simplest precursory technique for image segmentation. This methodology attempts to determine an intensity value that can separate the slice *g*(*x*, *y*) into two parts [15]. All voxels with intensities *f*(*x*, *y*) larger than the threshold value *T* are allocated into one class, and all the others into another class.

(7) $$\begin{array}{c} \text{g}\left(x,y\right)=\{\begin{array}{cc} f\left(x,y\right) & \text{if}\;f\left(x,y\right)>T,\\ 0 & \text{if}\;f\left(x,y\right)<T. \end{array} \end{array}$$

Thresholding approach does not consider the spatial characteristics of a volume; it is sensitive to noise and intensities variation. Thresholding approach has been used extensively in the literature as ground truth to compare some of the proposed schemes for medical image segmentation [16, 17]. 

#### 2.2.2. Soft Thresholding

Soft thresholding is more complex process compared to hard thresholding. This approach replaces each voxel which has a greater value than the threshold value by the difference between the threshold and the current voxel values. Soft thresholding could put into evidence some important regions as the region of interest (ROI) in this study. 

#### 2.2.3. Adaptive Thresholding

Otsu's method has been used as a third approach, which chooses the threshold that minimizes the intraclass variance of the black and white voxels in the volume [18]. Likewise, other variants of adaptive thresholding based on source-to-background ratio were also reported [4].

### 2.3. Multiresolution Analysis

Multiresolution analysis (MRA) is designed to give good time resolution and poor frequency resolution at high frequencies, and poor time resolution and good frequency resolution at low frequencies. It enables the exploitation of slice characteristics associated with a particular resolution level, which may not be detected using other analysis techniques [19–21]. The wavelet transform for a function *f*(*t*) can be defined as follows:

(8) $$\begin{array}{c} X_{\psi_{\left(a,b\right)}}=\overset{\infty }{\underset{-\infty }{\int }}f\left(t\right)\psi_{\left(a,b\right)}\left(t\right)dt, \end{array}$$

where

(9) $$\begin{array}{c} \psi_{\left(a,b\right)}\left(t\right)=\frac{1}{\sqrt{a}}\psi \left(\frac{t-b}{a}\right). \end{array}$$

The parameters *a*, *b* are called the scaling and shifting parameters, respectively [20, 22]. Haar wavelet filter will be used in the experimental study at different levels of decomposition. The Haar wavelet transform (HWT) of a two-dimensional slice can be performed using two approaches: the first one is called standard decomposition of a slice, where the one-dimensional HWT is applied to each row of voxel values followed by another one-dimensional HWT on the column of the processed slice. The other approach is called nonstandard decomposition, which alternates between the one-dimensional HWT operations on rows and columns. HWT serves as a prototype for all other wavelet transforms. Like all wavelet transforms, HWT decomposes a slice into four subimages of half the original size. HWT is conceptually simple, fast, memory efficient, and can be reversed without the edge effects that are associated with other wavelet transforms. HWT is a matrix-vector-based operation and can be formulated as follows:

(10) $$\begin{array}{c} H=\frac{1}{2}\left(\begin{array}{cc} 1 & 1\\ 1 & -1 \end{array}\right), \end{array}$$

(11) $$\begin{array}{c} O=I\times H, \end{array}$$

(12) $$\begin{array}{c} O={\left({\left(IH\right)}^{T}H\right)}^{T}=H^{T}IH, \end{array}$$

(13) $$\begin{array}{c} I={\left(H^{-1}\right)}^{T}OH^{-1}, \end{array}$$

where *I* is 2 × 2 input matrix, *H* contains the Haar coefficients, and *O* is the output matrix. Equations (12) and (13) show the transposed and reconstructed matrices, respectively. MRA has been used in the literature for different applications [22–24].

### 2.4. Clustering

Clustering techniques aim to classify each voxel in a volume into the proper cluster, then these clusters are mapped to display the segmented volume. The most commonly used clustering technique is the *K*-means method, which clusters *n* voxels into *K* clusters (*K* less than *n*) [25]. This algorithm chooses the number of clusters, *K*, then randomly generates *K* clusters and determines the cluster centers. The next step is assigning each point in the volume to the nearest cluster center, and finally recompute the new cluster centers. The two previous steps are repeated until the minimum variance criterion is achieved. This approach is similar to the expectation-maximization algorithm for Gaussian mixture in which they both attempt to find the centers of clusters in the volume. Its main objective is to achieve a minimum intracluster variance *V*

(14) $$\begin{array}{c} V=\overset{K}{\underset{i=1}{\sum }}\;\underset{x_{j}\in S_{i}}{\sum }{\left(x_{j}-\mu_{i}\right)}^{2}, \end{array}$$

where *K* is the number of clusters, *S* = 1,2,…, *K*, and *μ*_*i*_ is the mean of all voxels in the cluster *i*. *K*-means approach has been used with other techniques for clustering medical images [26].

## 3. Materials and Methods

### 3.1. The Proposed System

**Figure 1:**
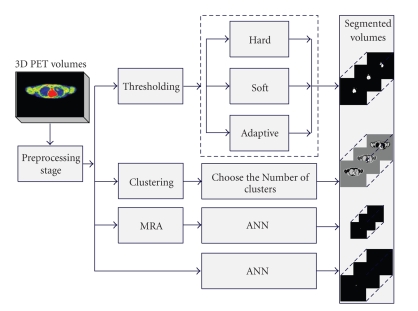
Proposed system for PET volume segmentation.

The proposed medical volume segmentation system is illustrated in Figure 1. The 3D PET volume acquired from the scanner goes through the preprocessing block, which enhances the quality of slice features and removes most of the noise from each slice. The enhanced volume can be processed using three approaches, the first processing block is thresholding which removes the background and unnecessary information producing a volume consists of two classes the background and the ROI. The second approach is *K*-means clustering technique which classifies each slice in PET volume into an appropriate number of clusters. The third approach is ANN which is used in both spatial and wavelet domains. The preprocessed PET volume is fed first to the ANN which is trained to detect the tumour. In another block, the PET volume is transformed into the wavelet domain using HWT at different levels of decomposition. This transform decomposes the volume and produces the approximation, horizontal, vertical, and diagonal features for each slice. The approximation features are fed to another ANN for classifying and quantifying the tumour. The outputs of ANNs are compared in the next step with the outputs of the other two approaches, while the best outputs are selected and displayed. The system has been tested using experimental and simulated phantom studies and clinical oncological PET volumes of nonsmall cell lung cancer patients.

### 3.2. Phantom Studies

In this study, PET volumes containing simulated tumour have been utilised. Two phantom data sets have been used. The first data set is obtained using NEMA IEC image quality body phantom which consists of an elliptical water-filled cavity with six spherical inserts suspended by plastic rods of volumes 0.5, 1.2, 2.6, 5.6, 11.5, and 26.5 ml (inner diameters of 10, 13, 17, 22, 28, and 37 mm). The voxel size is 4.07 mm × 4.07 mm × 5 mm, while the size of the obtained phantom volume is 168 × 168 × 66. This phantom was extensively used in the literature for assessment of image quality and validation of quantitative procedures [27–30]. Other variants of multisphere phantoms have also been suggested [31]. The PET scanner used for acquiring the data is the Biograph 16 PET/CT scanner (Siemens Medical Solution, Erlangen, Germany) operating in 3D mode [32]. Following Fourier rebinning and model-based scatter correction, PET images were reconstructed using two-dimensional iterative normalized attenuation-weighted ordered subsets expectation maximization (NAW-OSEM). CT-based attenuation correction was used to reconstruct the PET emission data. The default parameters used were ordered OSEM iterative reconstruction with four iterations and eight subsets followed by a postprocessing Gaussian filter (kernel full-width half-maximal height, 5 mm).

The second data set consists of Monte Carlo simulations of the Zubal antropommorphic model where two volumes were generated [33]. The first volume contains a matrix with isotropic voxels, the size of this volume is 128 × 128 × 180. The second volume contains the same matrix of the first one but with nonisotropic voxels having a matrix size of 128 × 128 × 375. The voxel size in both volumes is 5.0625 mm × 5.0625 mm × 2.4250 mm. The second data volume has 3 tumours in the lungs whose characteristics are given in Table 1. 

### 3.3. Clinical PET Studies

Clinical PET volumes of patients with histologically proven NSCLC (clinical Stage Ib-IIIb) who have undertaken a diagnostic whole-body PET/CT scan were used for assessment of the proposed segmentation technique [34]. Patients fasted no less than 6 hours before PET/CT scanning. The standard protocol involved intravenous injection of ^18^F-FDG followed by a physiologic saline (10 ml). The injected FDG activity was adjusted according to patient's weight using the following formula: A (Mbq) = weight (Kg) 4 + 20. After 45 min uptake time, free-breathing PET and CT images were acquired. The data were reconstructed using the same procedure described for the phantom studies. The maximal tumour diameters measured from the macroscopic examination of the surgical specimen served as ground truth for comparison with the maximum diameter estimated by the proposed segmentation technique. The voxel size is 5.31 mm × 5.31 mm × 5 mm, while the size of the obtained clinical volume is 128 × 128 × 178.

## 4. Results and Discussion

### 4.1. NEMA Image Quality Phantom

**Figure 2:**
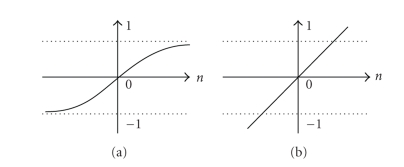
Activation functions: (a) Tangent-sigmoid function, (b) Linear function.

**Figure 3:**
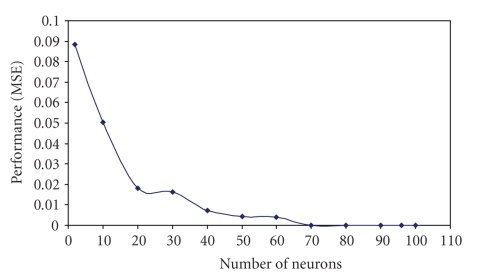
Evaluation of the number of neurons in the first hidden layer with neural network performance using MSE.

An experimental study has been run at the beginning to determine the best ANN design and algorithms. Multilayer feedforward NNs [8] consists of input layer (144 neurons), hidden layer (variant number of hidden neurons), and outputs layer (1) has been chosen first to determine the best number of hidden neurons. To evaluate the effect of the number of neurons in the hidden layer and achieve the best ANN performance for our application, different numbers of neurons in the hidden layer have been used. The maximum number of iterations used in the ANN is 1000. The experiment has been repeated 10 times for each chosen number of the hidden neurons, and the average was considered for that number. Hyperbolic tangent sigmoid transfer function has been used for all layers except the output layer where the linear activation function is used. The two activation functions are illustrated in Figure 2. Levenberg-Marquardt backpropagation training algorithm has been used during the evaluation of neurons numbers in the hidden layer [35] to validate the best design for the ANN, which is suitable for the proposed application. Figure 3 presents the number of neurons in the first hidden layer with the performance measured using mean-squared error (MSE) at 1000 iterations. The results obtained after this evaluation shows that the best number of the hidden neurons which corresponds to the smallest MSE, and good ANN outputs is 70 hidden neurons. 

Using the achieved ANN structure, different training algorithms have been evaluated in the next step to achieve the best ANN performance. In this evaluation the same ANN structure, sufficient training cases and 1000 epochs have been considered. The following training algorithms have been used in this part of the study. BFGS quasi-Newton backpropagation [36], bayesian regulation backpropagation (BR) [37], conjugate gradient backpropagation with Powell-Beale restarts (CGB) [38], conjugate gradient backpropagation with Fletcher-Reeves updates (CGF) [39], conjugate gradient backpropagation with Polak-Ribire updates (CGP) [39], gradient descent backpropagation (GD) [40], gradient descent with momentum backpropagation (GDM) [41], gradient descent with adaptive learning rate backpropagation (GDA) [42], gradient descent with momentum and adaptive learning rate backpropagation (GDX) [43], Levenberg-Marquardt backpropagation (LM) [35], one-step secant backpropagation (OSS) [44], random order incremental training with learning functions (R), resilient backpropagation (RP) [45], and scaled conjugate gradient backpropagation (SCG) [46]. The average of the performance and the required time for each of these training algorithms are presented in Table 2, this experiment has been repeated for 10 times and the standard deviation for the performance achieved is 4.15E-07. The best outputs associated with the best performance was achieved using Levenberg-Marquardt backpropagation training algorithm. This algorithm is using a combination of techniques which allows the NN to be trained efficiently. This combination includes backpropagation, gradient descent approach, and Gauss-Newton technique [47, 48].

After determining the main design parameters of ANN, a feedforward ANN with one hidden layer (70 hidden neurons), one outputs layer (1) has been used in the study of PET data sets. The training algorithm used with this network is Levenberg-Marquardt backpropagation algorithm. In this application, 70% of the first data set have been used for training (46 slices), 15% for validating (10 slices), and 15% for testing (10 slices). A window of 12 × 12 voxels has been used to scan each input slice. The size of this window is chosen to include all the spheres even the biggest one. The utilisation of this window has reduced the input features size fed into the ANN each time without losing the slice details in addition to reduce the required computational time. The input features of the ANN have been extracted in spatial and wavelet domains. For both domains an ANN with 144 inputs, 70 hidden neurons, and (1) outputs layer has been used. The input features in the spatial domain are the voxels of each processed slice. While the utilised wavelet filter decomposes each slice from the input volume and produces four types of coefficients. The approximation coefficients produced by the HWT represent the most detailed information about the analysed slice. The size of these coefficients (84 × 84) is half of the original size. The ANN achieved good performance with very small MSE, 2.39E-16.

**Figure 4:**
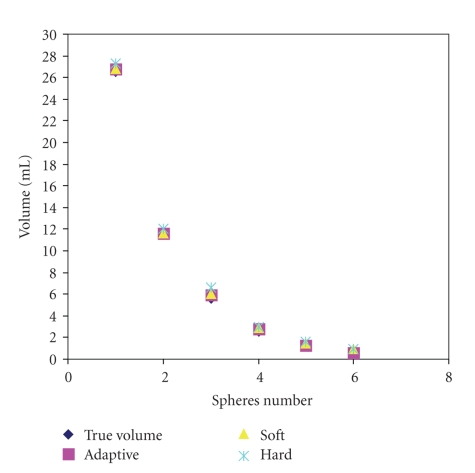
The segmented volume for all spheres in data set 1 using adaptive, soft, and hard thresholding approaches, respectively.

An objective evaluation of the artificial intelligence system (AIS) outputs has been performed by comparing the sphere computed volume (CV) with its true original volume (TV). The experimental results have been repeated 10 times, and the average of the sphere volume measured using ANN is calculated. The standard deviation for the volume of sphere 1 is 0.0971, for sphere 2 is 0.1170, for sphere 3 is 0.1185, for sphere 4 is 0.1232, for sphere 5 is 0.1258, and for sphere 6 is 0.1293. The CV obtained from the ANN, and the percentage of the absolute relative error (ARE %) for each sphere are presented in Table 3. ANN has clearly detected all spheres, where spheres 1, 2, and 3 are accurately segmented whereas spheres 4 is overestimated, and sphere 5 and 6 are underestimated. It is worth mentioning that the proposed system has shown better performance compared to the thresholding and clustering based approaches which are used as ground truth. Adaptive, soft, and hard thresholding approaches have been also used to perform the segmentation. The best results obtained from these approaches is by using adaptive threshold method which is used for the comparison with the other assessed techniques. Figure 4 presents the obtained spheres volumes using three thresholding approaches. Table 3 illustrates a comparison between the assessed approaches in term of ARE percentage. Thresholding approach has overestimated the volume of all spheres, while the exploitation of *K*-means clustering approach underestimates the volume of all spheres, particularly spheres 5 and 6.

**Figure 5:**
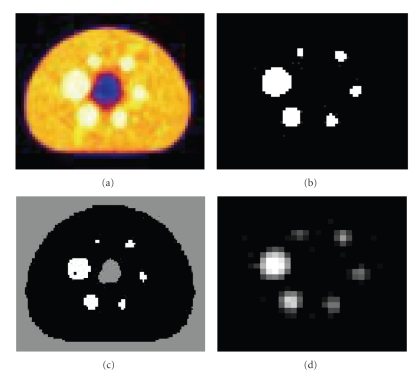
Phantom data set 1: (a) Original PET image (168 × 168), (b) thresholded image (168 × 168), (C) clustered image (168 × 168), (d) segmented image (84 × 84) using ANN and MRA, zoomed by a factor of 2.

**Figure 6:**
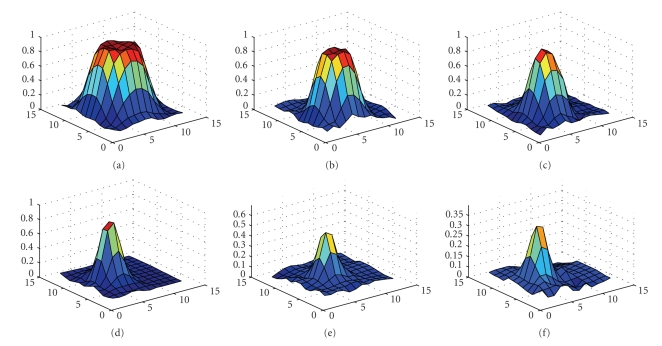
Segmented sphere surface plot for phantom data set 1. Voxel values scaled in [0..1] on Z axis, and voxels number is within [0..12] on X and Y axes: (a) sphere 1, (b) sphere 2, (c) sphere 3, (d) sphere 4, (e) sphere 5, and (f) sphere 6.

The segmented slices from thresholding, clustering and ANN in the wavelet domain are illustrated in Figure 5, where Figure 5(d) is zoomed for illustration purpose. The three-dimensional shaded surface for each segmented sphere obtained from ANN are plotted in Figure 6, where the voxel values are scaled in [0..1] on Z axis and voxels number is within [0..12] on the remaining two axes.

### 4.2. Simulated Zubal Phantom

**Figure 7:**
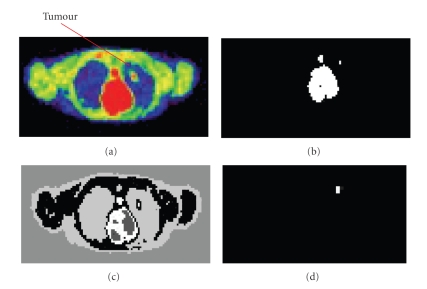
Phantom data set 2. (Tumour 1): (a) Original PET image (128 × 128), (b) thresholded image (128 × 128), (C) clustered image (128 × 128), (d) segmented image (64 × 64) using ANN and MRA, zoomed by a factor of 2, where tumour 1 (2 voxel) is detected.

The proposed segmentation system was able to detect tumours in the second phantom data set with isotropic voxels. The first tumour with size of 2 voxels was clearly detected in slice 68. Figure 7 shows the segmented slices from thresholding, clustering, and ANN in the wavelet domain for this tumour. The second and third tumours with size 3 and 2 voxels, respectively, were also clearly detected in slice 57 and 74, respectively. Similar results have been achieved for detecting tumours in the second data set with nonisotropic voxels. On the other hand similar segmented slices have been obtained using ANN in the spatial domain, however, more computational time is required for processing all data sets in this domain. 

### 4.3. Performance Evaluation

**Figure 8:**
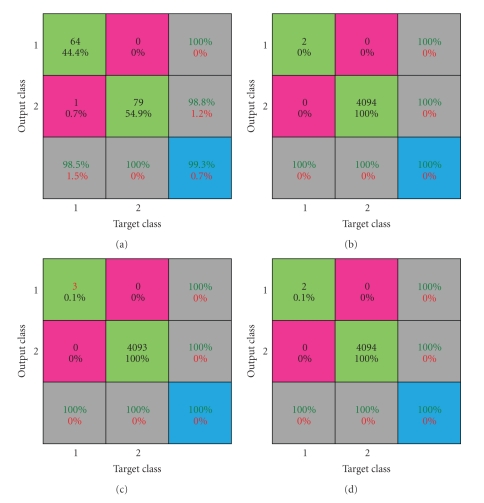
Confusion matrix: (a) for phantom data set 1, sphere 1, (b) phantom data set 2, tumour 1, (c) phantom data set 2, tumour 2, and (d) for phantom data set 2, tumour 3.

In the field of AI a number of performance metrics can be employed to evaluate the performance of ANN. A confusion matrix is a visualisation tool typically used in supervised and unsupervised learning approaches. Each row of the matrix represents the instances in a predicted class, while each column represents the instances in an actual class. One benefit of a confusion matrix is that it is easy to see if the system is confusing two classes (the tumour and the remaining tissues). The confusion matrix for the first data set shows that 1 voxel out of 65 ones in the first segmented sphere was misclassified, Figure 8(a). Where the percent in the green box refers to each class prediction accuracy. While the percent in the pink box refers to the misclassified voxels accuracy in each class. The gray boxes represent the percents of classified voxels numbers in each class in green, and the percent of the error in each class in red. The blue box represents the total percent of all classes in green and the total error in these classes in red. All the numbers in the confusion matrix are represented as a percentage.

The confusion matrix for the second data set, tumour 1 is illustrated in Figure 8(b). The two voxels of this tumour were precisely classified in one class and the remaining voxels (4094) classified in the other class. The confusion matrix for the second data set, tumour 2, shows that the 3 voxels were precisely classified as a first class and the remaining voxels (4093) classified in the other class. The obtained result is presented in Figure 8(c), while Figure 8(d) illustrates the confusion matrix for the second data set, tumour 3. The two voxels of tumour 3 were precisely classified as a first class and the remaining voxels (4094) classified in the other class.

The other performance checking approach is receiver operating characteristic (ROC). This approach can be represented by plotting the fraction of true positives rate (TPR) versus the fraction of false positives rate (FPR), where the perfect point in the ROC curve is the point (0,1). The ROC curve for the first data set is located near the perfect point and the FPR for the sphere voxels number is near the 0 point. Perfect ROC has been obtained for the second data set and the FPR for tumour voxels number is 0.

### 4.4. Clinical PET Studies

**Figure 9:**
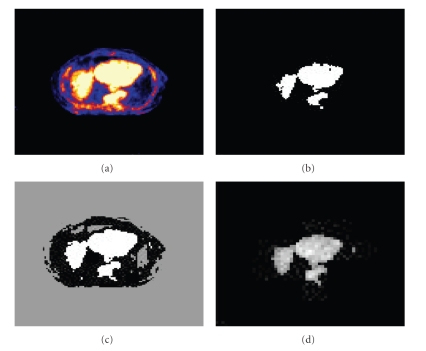
Illustration of algorithm performance on clinical PET data showing: (a) original PET image (128 × 128), (b) thresholded image (128 × 128), (C) clustered image (128 × 128), and (d) segmented image (64 × 64) using ANN and MRA, zoomed by a factor of 2.

The proposed approaches have been also tested on clinical PET volumes of nonsmall cell lung cancer patients. A subjective evaluation based on the clinical knowledge has been carried out for the output of the proposed approaches. The tumour in these slices has a maximum diameter on the *y*-axis of 90 mm (estimated by histology). The segmented tumour using ANN in spatial domain and wavelet domain (after scaling) has a diameter of 90.1 mm. The segmented volumes using the proposed approaches outlines a well defined contour as illustrated in Figure 9.

## 5. Conclusions

An artificial intelligence system based on multilayer artificial neural networks was proposed for PET volume segmentation. Different training algorithms have been utilised in this study to validate the best algorithm for the targeted application. Two PET phantom data sets and a clinical PET volume of nonsmall cell lung cancer patient have been used to evaluate the performance of the proposed system. Objective and subjective evaluation for the system outputs have been carried out. Confusion matrix and receiver operating characteristic were also used to judge the performance of the trained neural network. Experimental and simulated phantom results have shown a good performance for the ANN in detecting the tumours in spatial and wavelet domains for both phantom and clinical PET volumes. Accurate tumour quantification was also achieved through this system. Ongoing research is focusing on further validation of the proposed algorithm in a clinical setting and the exploitation of other artificial intelligence tools and feature extraction techniques.

## Figures and Tables

**Table 1:** Tumours characteristics for the second data set.

Tumour	Isotropic		Nonisotropic	
Number	Position	Size	Position	Size
1	slice 68	2 voxels	slice 142	2 voxels
2	slice 57	3 voxels	slice 119	3 voxels
3	slice 74	2 voxels	slice 155	2 voxels

**Table 2:** Evaluation of the effect of different training algorithms on the performance of multilayer feedforward NN with 144-70-1 topology.

Training function	Time (sec)	Performance (MSE)
BFG	58	0.000426
BR	35	3.69e-6
CGB	11	0.0128
CGF	11	0.0156
CGP	13	0.0160
GD	6	0.117
GDM	6	0.100
GDA	6	0.0950
GDX	6	0.0383
LM	27	1.32E-7
OSS	13	0.0198
R	226	0.0503
RP	6	0.0183
SCG	10	0.0154

**Table 3:** Comparison of sphere volumes and ARE between TV and CV for different segmentation algorithms assessed including thresholding, clustering and ANN.

Spheres		Thresholding		Clustering		ANN	
No.	TV (ml)	CV (ml)	ARE %	CV (ml)	ARE %	CV (ml)	ARE %
1	26.52	26.74	0.81	25.73	2.97	26.54	0.08
2	11.49	11.62	1.07	11.01	4.22	11.51	0.10
3	5.58	5.96	6.97	5.28	5.35	5.63	0.95
4	2.57	2.81	9.08	2.40	6.89	2.63	2.13
5	1.15	1.29	11.98	1.03	10.03	1.10	4.54
6	0.52	0.61	15.76	0.46	11.52	0.49	5.88
